# A prospective, multicenter phase I/II study of induction chemotherapy with docetaxel, cisplatin and fluorouracil (DCF) followed by chemoradiotherapy in patients with unresectable locally advanced esophageal carcinoma

**DOI:** 10.1007/s00280-016-3062-2

**Published:** 2016-05-18

**Authors:** Hironaga Satake, Makoto Tahara, Satoshi Mochizuki, Ken Kato, Hiroki Hara, Tomoya Yokota, Naomi Kiyota, Takayuki Kii, Keisho Chin, Sadamoto Zenda, Takashi Kojima, Hideaki Bando, Tomoko Yamazaki, Satoru Iwasa, Yoshitaka Honma, Satoru Hamauchi, Takahiro Tsushima, Atsushi Ohtsu

**Affiliations:** Department of GI Oncology, National Cancer Center Hospital East, Kashiwa, Japan; Department of Medical Oncology, Kobe City Medical Center General Hospital, Kobe, Japan; Department of Head and Neck Medical Oncology, National Cancer Center Hospital East, 6-5-1, Kashiwanoha, Kashiwa, 277-8577 Japan; Gastroenterology Division, Tokatsu-Tsujinaka Hospital, Abiko, Japan; Gastrointestinal Medical Oncology Division, National Cancer Center Hospital, Tokyo, Japan; Department of Gastroenterology, Saitama Cancer Center, Saitama, Japan; Division of Gastrointestinal Oncology, Shizuoka Cancer Center, Shizuoka, Japan; Department of Oncology, Kobe University Hospital, Kobe, Japan; Department of Cancer Chemotherapy Center, Osaka Medical College, Osaka, Japan; Department of Gastroenterology, Cancer Institute Hospital of the Japanese Foundation for Cancer Research, Tokyo, Japan; Division of Radiation Oncology, National Cancer Center Hospital East, Kashiwa, Japan

**Keywords:** Esophageal carcinoma, Squamous cell carcinoma, Induction chemotherapy, Chemoradiotherapy, DCF

## Abstract

**Purpose:**

Standard care for unresectable locally advanced esophageal squamous cell carcinoma (ESCC) is concurrent chemoradiotherapy, but survival remains limited. Neoadjuvant chemotherapy with docetaxel, cisplatin and fluorouracil (DCF) has demonstrated promising activity, with a pathological complete response (CR) of 17 % for resectable stage II/III ESCC. Here, we conducted a multicenter study to assess the efficacy and safety of induction chemotherapy with DCF followed by CRT in patients with unresectable locally advanced ESCC.

**Methods:**

Eligibility criteria included clinical T4 and/or M1 lymph node ESCC, PS 0–1 and age 20–70 years. Treatment consisted of docetaxel 70 mg/m^2^ and cisplatin 70 mg/m^2^ on day 1, and fluorouracil 750 mg/m^2^ on days 1–5, repeated every 3 weeks for three cycles, followed by cisplatin 70 mg/m^2^ on days 64 and 92, and fluorouracil 700 mg/m^2^ on days 64–67 and 92–95, concurrently with radiotherapy (60 Gy in 30 fractions, 5 days/week). Primary endpoint of the phase II part was CR rate.

**Results:**

Thirty-three patients were enrolled. The completion rate of protocol treatment was 88 %. Thirteen patients (39.4 %) achieved a CR. With a median follow-up period of 41 months (range 24–49 months), median progression-free survival was 12.2 months, and median survival was 26.0 months, with a survival rate of 40.4 % at 3 years. The most common grade 3 or 4 toxicities were neutropenia, leukopenia, anorexia and dysphagia. No treatment-related death was observed.

**Conclusion:**

Induction chemotherapy with DCF followed by CRT is tolerable and shows promising efficacy for unresectable locally advanced ESCC.

## Introduction

Esophageal squamous cell carcinoma (ESCC) is a highly malignant disease. The high frequency of unresectable primary disease, distant metastases or medical unsuitability for surgery at initial diagnosis means that 40–60 % of patients are not candidates for surgery, and their prognosis remains dismal [1]. Curative resection is not feasible in patients with locally advanced ESCC, particularly those with direct invasion of adjacent organs T4, and such cases have an unfavorable prognosis [2–4]. Standard care for unresectable locally advanced ESCC is concurrent chemoradiotherapy (CRT). A combination of cisplatin and fluorouracil (CF) has become the standard regimen, but survival remains poor [5–7]. A previous study of CRT with CF plus 60 Gy of radiation in patients who had ESCC with T4 tumors and/or M1 lymph node metastasis (M1 LYM) showed a CR rate of 15–33 % and 3-year overall survival rate of 23–26 % [3, 8, 9].

Docetaxel has shown activity against many solid tumors as monotherapy and in combination with other agents. Docetaxel has a different mechanism of action to CF and has been proved to have an additive effect with cisplatin and supra-additive antitumor activity with fluorouracil in vitro and in murine models of xenografted human tumors [10, 11]. Two phase III trials showed survival benefits from induction chemotherapy with docetaxel plus cisplatin and fluorouracil (DCF) compared to CF in locally advanced head and neck squamous cell carcinoma [12, 13]. Neoadjuvant chemotherapy with DCF demonstrated promising activity with a pathological CR rate of 17 % for resectable stage II/III ESCC [14]. Results were also promising in a phase II trial of DCF followed by carboplatin and radiotherapy in locally advanced ESCC [15]. Furthermore, the risk of perforation of the esophageal wall related to definitive CRT has been highlighted in patients with T4 disease. To reduce the risk of perforation, we decided to use induction chemotherapy before CRT, with the aim of decreasing tumor volume before encountering severe esophagitis. However, the efficacy and safety of induction DCF followed by CF-RT for ESCC with T4 tumors and/or M1 lymph node metastasis (M1 LYM) has not been reported.

In this study, we aimed to assess the efficacy and safety of induction chemotherapy with DCF followed by CRT in patients with unresectable locally advanced ESCC.

## Materials and methods

### Eligibility criteria

Eligibility criteria were age 20–70 years; histologically proven squamous cell carcinoma of the esophagus; primary lesion located within the thoracic esophagus; no distant organ metastases; no esophagobronchial or esophagomediastinal fistulas; Eastern Cooperative Oncology Group (ECOG) performance status (PS) <2; adequate organ function defined by hemoglobin ≥10 g/dl, absolute neutrophil count ≥2×10^9^/l, platelet count ≥100 × 10^9^/l, total bilirubin ≤1.5 mg/dl and serum transaminases ≤3 × the upper normal limit (UNL) of the institution and creatinine clearance ≥60 mL/min, PaO_2_ ≥70 mmHg; and tumors judged unresectable by computed tomography (CT) scan defined by (1) primary tumor invasion depth T4 and/or (2) metastatic regional lymph node invasion to adjacent organs and/or (3) M1 lymph node (M1 LYM). T4 was defined as a tumor that invades contiguous structures and M1 LYM as nodal metastasis beyond the regional lymph nodes, such as the supraclavicular or celiac lymph nodes (International Union Against Cancer TNM classification system, sixth edition). All areas of nodal disease had to be encompassable within the radiation field. Exclusion criteria were history of prior chemotherapy; myocardial infarction within the last 3 months; history of unstable angina pectoris, intestinal pneumonia, fibroid lung or severe emphysema; concurrent active malignancy; uncontrolled infection; and pregnancy or lactation. All patients were required to provide written informed consent before entering the study, which was approved by the institutional review board at each participating center.

### Study design and treatment

The concurrent phase I part of the study was conducted in two cancer centers, and the subsequent phase II part was conducted in seven referral centers. Protocol treatment was defined as induction chemotherapy consisting of docetaxel (DTX), cisplatin (CDDP) plus fluorouracil (5-FU) (DCF) followed by chemoradiotherapy concurrent with CDDP plus 5-FU. Induction chemotherapy consisted of a 1-h intravenous (i.v.) administration of DTX on day 1; 2-h infusion of CDDP on day 1; and continuous i.v. administration of 5-FU on days 1–5. This regimen was repeated every 3 weeks for a maximum of up to three cycles. Chemoradiotherapy consisted of concurrent administration of CDDP (70 mg/m^2^ on days 64 and 92) plus 5-FU (700 mg/m^2^ on days 64–67 and 92–95) with radiotherapy.

Radiotherapy consisted of 60 Gy with a daily dose of 2 Gy and was delivered with ≥6-MV X-rays. Three-dimensional treatment planning with a CT stimulator was used. Gross tumor volume (GTV) was determined by pretreatment with CT and GI endoscopy. Clinical target volume (CTV) included GTV with a craniocaudal margin of 3 cm in the primary site and a margin of 0–1 cm in lymph node metastases. Because target volume is always large in far-advanced esophageal cancer, no prophylactic irradiation for lymph node areas was performed. Completion of protocol treatment was defined as the end of induction DCF followed by concurrent CF-RT consisting of 60 Gy within 24 weeks from the date of first administration of induction DCF.

Prophylactic use of granulocyte colony-stimulating factor (G-CSF) was not allowed, but ciprofloxacin was administered on days 5–15.

The phase I part was designed to determine the recommended dose (RD) of induction chemotherapy. Six patients were treated at dose level 1 (DTX 70 mg/m^2^, CDDP 70 mg/m^2^ and 5-FU 750 mg/m^2^). If three or more of the six patients experienced a dose-limiting toxicity (DLT), six additional patients were accrued at the next lower dose level. The RD was defined as the dose at which two or fewer of six patients experienced a DLT. If two or fewer of the six patients in dose level 1 experienced a DLT, the RD was determined to be level 1 (DTX 70 mg/m^2^, CDDP 70 mg/m^2^ and 5-FU 750 mg/m^2^).

The dose was modified for each patient based on hematologic or non-hematologic toxicity. DLT was defined as any of the following adverse events occurring within 28 days after completion of the protocol treatment: (1) febrile neutropenia lasting >4 days; (2) grade 4 thrombocytopenia (<0.25 × 10^9^/l); (3) grade 3 or 4 non-hematologic toxic effects, except grade 3 alopecia, anorexia, nausea, vomiting, constipation, stomatitis, esophagitis or infection due to stomatitis; (4) discontinuation of treatment due to an adverse event; or (5) treatment-related death.

In the subsequent phase II part, the enrolled patients were treated with the RD of induction chemotherapy followed by concurrent chemoradiotherapy as above.

### Study assessment

Pretreatment evaluation included a medical history; physical examination; complete blood cell count and serum chemistry tests; esophagogastroduodenoscopy; and cervical, chest and abdominal CT scans. Endoscopic ultrasound, bronchoscopy and cervical ultrasound were optional. Adjacent organs were considered to be involved if the tumor extended into the lumen or caused a deformity of the airway in the trachea or tracheobronchial tree, and if the tumor was attached to the organ at a contact angle ≥90° in the thoracic aorta as observed on the CT scan. T3 or lesser extent of disease was determined by endoscopic ultrasound. Lymph nodes were considered positive if they were ≥1 cm in diameter on any image. These evaluations for staging were reviewed, and cases were judged as potentially incurable with surgery by diagnostic radiologists as well as surgeons and medical oncologists at each institution.

All adverse events experienced during the study were recorded and graded according to the National Cancer Institute Common Terminology Criteria for Adverse Events (CTCAE version 3.0). Late toxicity was graded according to the RTOG/EORTC Late Radiation Morbidity Scoring Scheme. Late toxicity was defined as toxicity occurring more than 31 days after treatment completion. Close follow-up using both endoscopy and CT was mandatory in the third week of every induction chemotherapy administration. If disease progression or new metastasis was detected, the subsequent cycle of induction chemotherapy was discontinued and a shift to chemoradiation was mandated. A history and physical examination, serum chemistry profile, cervical–chest–abdominal CT scan and esophagogastroduodenoscopy were performed in the fourth week after the completion of all protocol therapy. The following were performed every 3 months for 1 year and every 6 months thereafter until disease progression: physical examination, toxicity assessment, complete blood cell count, serum chemistry profile, cervical–chest–abdominal CT scan and esophagogastroduodenoscopy. Patterns of failure were defined as the first site of failure. Local/regional failure included the primary tumor and regional lymph nodes. Distant failure included any site beyond the primary tumor and regional lymph nodes.

### Endpoints and statistical analysis

Once the RD of induction chemotherapy was determined in the first phase of the study, patient accrual continued for the phase II study, the main objective of which was to determine the objective response activity of this strategy. The primary endpoint was complete response (CR) rate evaluated according to the Response Evaluation Criteria in Solid Tumors (RECIST) criteria v1.0 and endoscopic assessment for the primary tumor. Primary tumor response was evaluated using endoscopy in accordance with the modified criteria of the tenth edition of Guidelines for the Diagnosis and Treatment of Carcinoma of the Esophagus, issued by the Japanese Society for Esophageal Diseases [16]. For the primary site, clinical CR was defined as disappearance of the primary tumor, ulceration and erosion as confirmed by endoscopic examination and negative biopsy results. A CR of lymph node metastasis was defined as the disappearance of all visible lymph node metastases on CT imaging. A CR was defined as a clinical CR of the primary tumor and CR of lymph node metastases. An evaluation of CR had to be confirmed by reassessment on endoscopy and CT 4 or more weeks later. Secondary endpoints included progression-free survival (PFS), overall survival (OS), response rate of induction chemotherapy, completion rate of protocol treatment and safety. We set the threshold objective CR rate at 30 % and the expected objective CR rate at 50 % on the basis of the results of previous studies [3, 8]. Given a one-sided α of 0.1 and statistical power of 80 %, a minimum of 27 patients was required.

The survival curve was estimated using the Kaplan–Meier method. Safety and efficacy analyses were both conducted on an intention-to-treat (ITT) population, defined as all patients enrolled in the study who received at least one dose of induction chemotherapy. The PFS was defined as the time from the date of first administration of induction chemotherapy to the first documentation of disease progression, subsequent therapy or death. OS was determined from the date of first administration of induction chemotherapy to the date of death or last confirmation of survival. Statistical data were obtained using the SPSS software package (SPSS 22.0 Inc., Chicago, IL).

This trial was registered with University Hospital Medical Information Network (No. UMIN000003370).

## Results

### Patient characteristics

Thirty-three patients with histologically proven squamous cell carcinoma were enrolled from August 2009 to November 2011. The 33 patients are characterized in Table 1. Baseline nutritional statuses were total protein median 7.0 g/dl (range 5.9–8.0) and serum albumin level median 4.0 g/dl (range 2.8–4.8). There were 16 patients (48 %) with T4 M0 disease, 13 (39 %) with non-T4 M1 LYM and 4 (12 %) with T4 M1 LYM. The site of clinical involvement in the 20 cases of T4 disease was the trachea in 15, trachea and thoracic aorta in 2 and thoracic aorta, pericardium and stomach in 1 case each.

**Table 1:** Patient characteristics (*n* = 33)

Variable		*n* (%)
Age	Median	61
	Range	30–69
Sex	Male	29 (88)
	Female	4 (12)
ECOG PS	0	19 (58)
	1	14 (42)
Tumor location	Upper thorax	18 (55)
	Middle thorax	13 (39)
	Lower thorax	2 (6)
Clinical TNM and stage		
III	T4N1M0	16 (48)
IVA	T2N0M1a	1 (3)
	T2N1M1a	1 (3)
	T3N1M1a	8
	T4N1M1a	3
IVB	T3N1M1b	3
	T4N1M1b	1 (3)

*ECOG* Eastern Cooperative Oncology Group, *PS* performance status

Among the six patients who were registered to phase I, all patients were treated at the RD (level 1: DTX 70 mg/m^2^, CDDP 70 mg/m^2^ and 5-FU 750 mg/m^2^). Twenty-seven other patients were entered into phase II to further evaluate the tolerability and toxicity of the study regimen. All 33 patients were evaluated for toxicity and efficacy.

### Phase 1

The first six patients were enrolled at dose level 1 (DTX 70 mg/m^2^, CDDP 70 mg/m^2^ and 5-FU 750 mg/m^2^). No DLTs were observed, and hence, the RD was determined to be DTX 70 mg/m^2^, CDDP 70 mg/m^2^ and 5-FU 750 mg/m^2^. All six patients completed the three courses of induction chemotherapy, and five patients received subsequent chemoradiation without cessation. The sixth patient required the temporary cessation of radiation therapy due to infection for 5 days, but completed all subsequent irradiation.

### Toxicity

The worst toxicity throughout the treatment period is listed in Table 2. During the induction chemotherapy phase, grade 3 or 4 neutropenia and febrile neutropenia (FN) occurred in 72 and 6 % of patients, respectively. Of these patients, 12 patients were administered G-CSF for FN or grade 4 neutropenia. One patient with grade 4 FN developed pneumonia with shock, but recovered following treatment with G-CSF and antibiotics. Another patient with grade 4 FN, whose primary tumor involved the trachea at initial diagnosis, developed treatment-related perforation of the esophageal wall, leading to an esophagotracheal fistula after the first cycle of induction chemotherapy.

**Table 2:** Adverse events during the treatment period (*n* = 33)

	Grade					
	1	2	3	4	All, %	3/4, %
*Adverse events during induction chemotherapy*						
Hematologic						
Leukopenia	5	15	11	2	100	39
Neutropenia	0	9	12	12	100	72
Anemia	22	11	0	0	100	0
Thrombocytopenia	22	11	0	0	100	0
Non-hematologic						
Creatinine increased	7	0	0	0	21	0
Elevation of AST	14	0	0	0	42	0
Elevation of ALT	10	9	1	0	30	3
Febrile neutropenia	–	–	1	1	6	6
Hyperbilirubinemia	3	1	0	0	12	0
Hyponatremia (≥G3)	–	–	1	1	6	6
Alopecia	14	6	0	0	60	0
Anorexia	10	13	6	0	87	18
Constipation	8	6	0	0	42	0
Diarrhea	9	3	0	0	38	0
Dysphagia	7	6	4	0	51	12
Edema	2	0	1	0	9	3
Fever	2	0	1	0	9	3
Fistula GI-esophagus	0	0	1	0	3	3
Infection with normal ANC	0	0	1	1	6	6
Nausea	13	7	3	0	69	9
Rash	3	0	0	0	9	0
Stomatitis	11	6	1	0	54	3
Vomiting	3	2	0	0	15	0
*Adverse events during chemoradiation*						
Hematologic						
Leukopenia	5	15	8	0	85	24
Neutropenia	11	10	6	0	82	18
Anemia	6	22	1	0	88	3
Thrombocytopenia	9	2	0	0	33	0
Non-hematologic						
Creatinine increased	8	1	0	0	27	0
Elevation of AST	5	0	0	0	15	0
Elevation of ALT	0	7	0	0	21	0
Febrile neutropenia	–	–	1	0	3	3
Alopecia	14	8	0	0	67	0
Anorexia	12	6	4	0	67	12
Constipation	9	0	0	0	27	0
Diarrhea	4	0	0	0	12	0
Dysphagia	8	8	5	0	64	15
Edema	1	1	0	0	6	0
Esophagitis (≥G3)	–	–	4	0	–	12
Fever	2	0	0	0	6	0
Fistula GI-esophagus	0	0	1	0	3	3
Infection with normal ANC	1	0	1	0	6	3
Nausea	12	3	3	0	55	9
Neuropathy sensory	2	0	0	0	6	0
Stomatitis	4	0	0	0	12	0
Vomiting	3	1	0	0	12	0

During the chemoradiation phase, grade 3 or 4 neutropenia and FN occurred in 18 and 3 % of patients, respectively. Of these, one patient required G-CSF for 2 days starting from the 20th day of irradiation. One patient with T4 disease involving the trachea at initial diagnosis developed treatment-related perforation of the esophageal wall, and an esophagomediastinal fistula occurred on the 16th day from the start of irradiation. No treatment-related death was observed during the protocol treatment.

Six patients experienced late toxicities related to treatment, two with grade 2 esophageal stricture, two with grade 1 or 2 peripheral sensory neuropathy, one with grade 1 pleural effusion and one with grade 1 radiation pneumonitis. No patient experienced grade 3 or higher late toxicity.

### Dose intensity

During the induction chemotherapy phase, the median percentage of relative dose intensity (RDI) delivered was 83.6 % (range 33.0–100) for docetaxel, 81.5 % (range 33.0–100) for cisplatin and 84.1 % (range 33.2–100) for fluorouracil. The average RDI of induction chemotherapy was 83.0 %. Four of 33 patients (12 %) discontinued induction chemotherapy due to toxicity; of these, 3 discontinued after the first cycle of induction DCF, one each due to grade 4 hyponatremia, grade 4 febrile neutropenia and esophagotracheal fistula. One patient discontinued induction DCF after the second cycle due to grade 4 sepsis. During the chemoradiation phase, the median dose intensity delivery was 85.8 % (range 30.0–100) for cisplatin and 94.1 % (range 49.9–100) for fluorouracil. The average RDI of chemotherapy was 90.0 %. Patients required dose reduction mainly due to bone marrow suppression, renal toxicity (creatinine clearance < 60 mL/min) or stomatitis. The completion rate of protocol treatment was 87.8 %.

### Response to treatment

Response to induction chemotherapy and chemoradiotherapy is summarized in Table 3. Two of the 33 patients had CR after induction chemotherapy, and thirteen patients (39.4 %, 95 % CI 21.8–57.0 %) had CR after chemoradiation.

**Table 3:** Response rate to treatment (*n* = 33)

Variable	*n*	% (95% CI)
Response after induction chemotherapy		
Overall	20	60.6 (43.7–77.5)
Complete response	2	6.1 (0–14.7)
Partial response	18	54.5 (36.6–72.5)
Response after CRT		
Overall	24	72.7 (55.8–84.9)
Complete response	13	39.4 (24.7–56.3)
Partial response	11	33.3 (19.8–50.4)

*CI* confidence interval, *CRT* chemoradiation

### Survival and patterns of failure

**Fig. 1:**
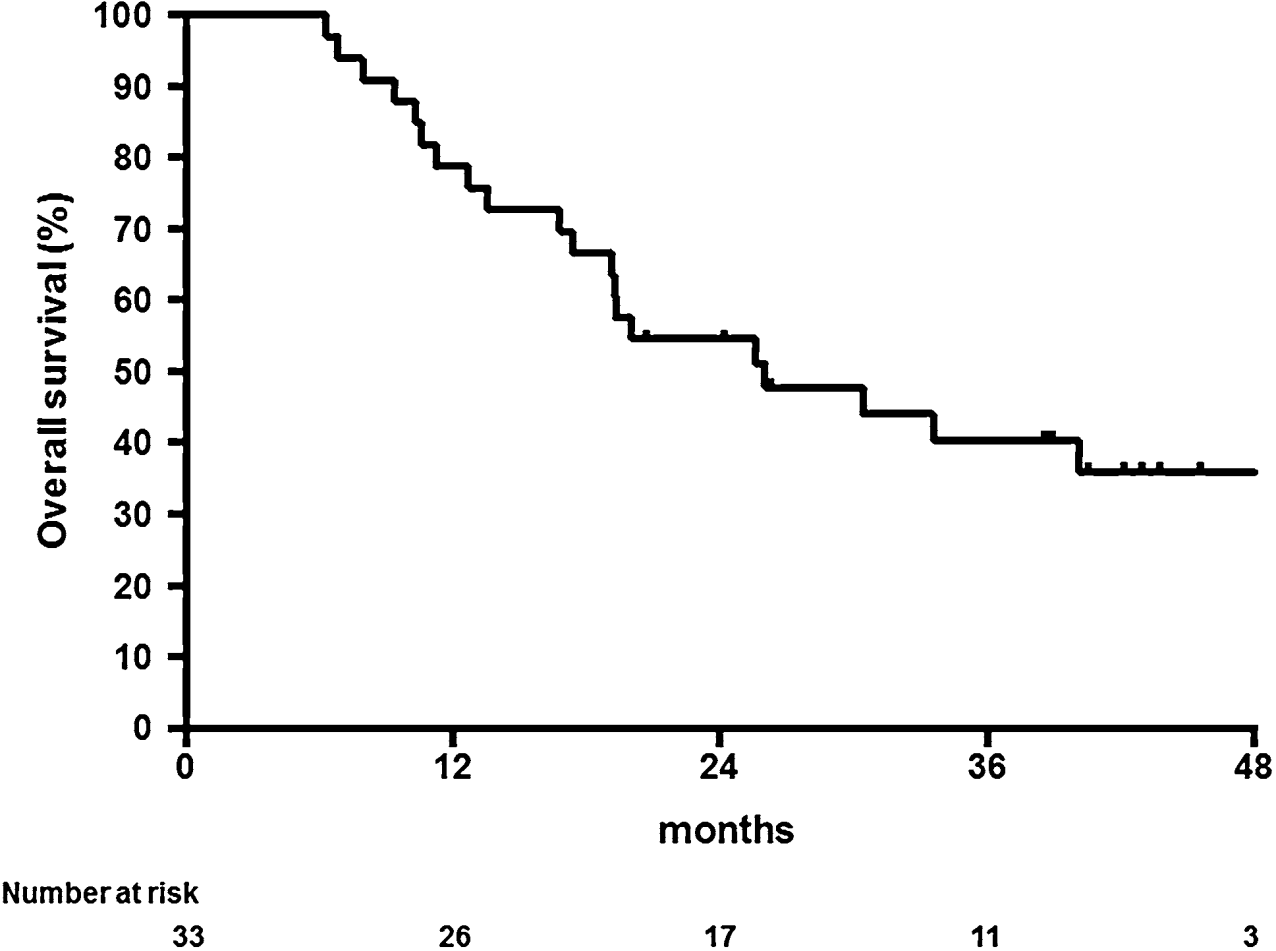
Kaplan–Meier estimates of overall survival (*n* = 33)

**Fig. 2:**
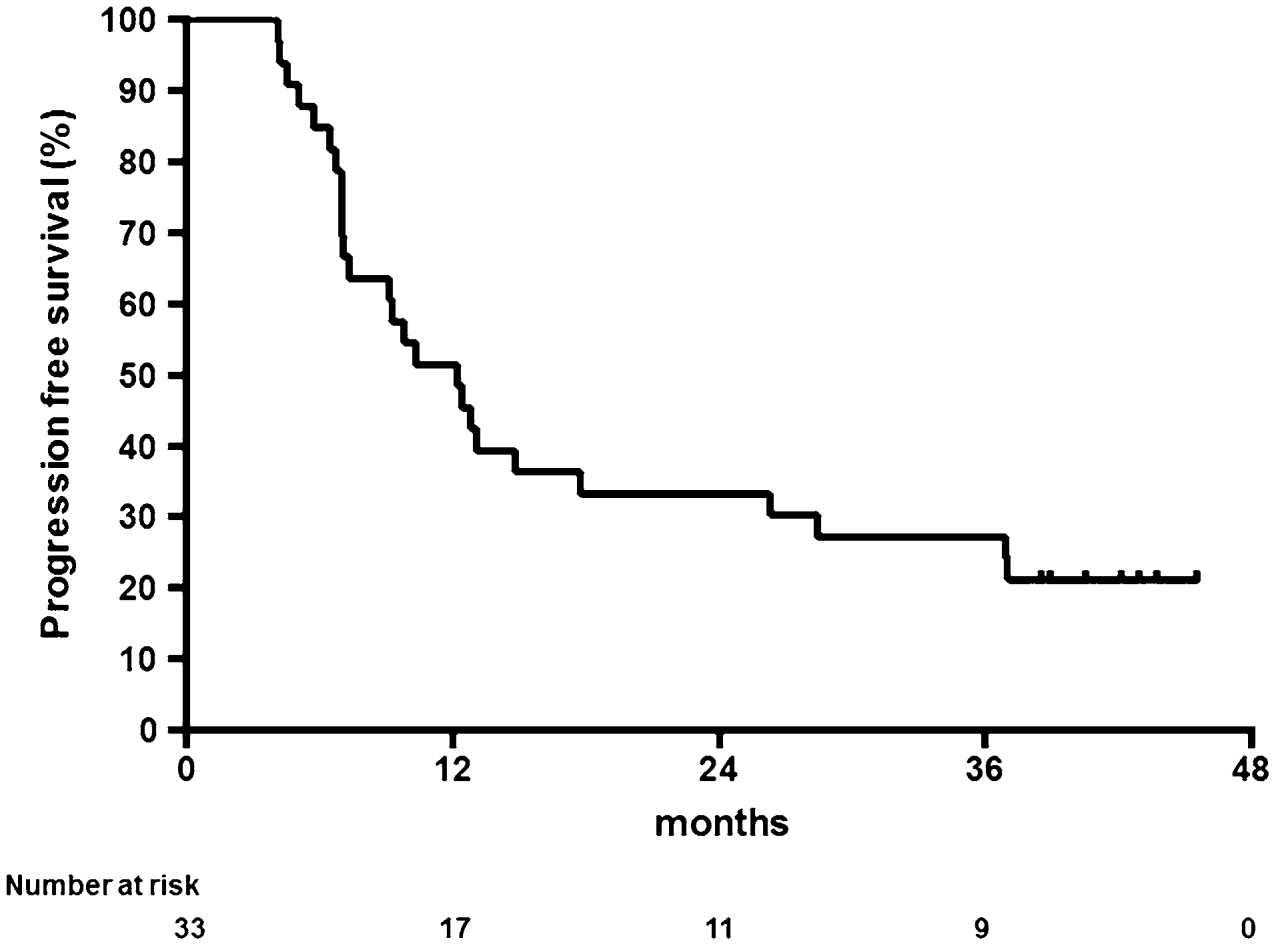
Kaplan–Meier estimates of progression-free survival (*n* = 33)

With a median follow-up period of 41 months (range 24–49 months), median survival was 26.0 months (95 % CI 11.8–40.2), and 1- and 3-year survival rates were 78.8 % (95 % CI 60.6–89.3) and 40.4 % (95 % CI 23.3–56.9), respectively (Fig. 1). The median time to progression was 12.2 months (95 % CI 8.2–16.2), and 1- and 3-year progression-free survival rates were 51.5 % (95 % CI 33.5–66.9) and 27.3 % (95 % CI 13.6–42.9), respectively (Fig. 2). No significant difference (*p* = 0.911) was seen in survival benefit between T4 and non-T4 groups, with an overall survival time of 26.0 versus 25.6 months, respectively.

Among all 33 patients, 21 (64 %) received post-protocol treatment. Eight patients underwent salvage surgery, with five cases of curative resection. Three patients underwent salvage endoscopic resection, with two cases of pathological complete resection. Three cases went off-protocol due to adverse events during induction chemotherapy and received definitive chemoradiotherapy (two with CF-RT and one with 5-FU/nedaplatin-RT), with one case of complete response. One patient received definitive chemoradiotherapy with a CF regimen due to recurrence out of a prior radiation field. Nine patients received palliative chemotherapy for progression.

Patterns of first failure are shown in Table 4. All failures occurred within 1 year (range 6.1–8.0 months) after confirmation of clinical CR.

**Table 4:** Patterns of failure (*n* = 33)

	*n*	%
Alive/no failure	8	24
Any failure	25	76
Persistent local disease	18	55
Local recurrence	1	3
Regional lymph node recurrence	3	9
Distant recurrence	3	9

## Discussion

In this multicenter prospective trial of induction chemotherapy followed by concurrent CRT, induction DCF showed substantial activity in patients with unresectable locally advanced ESCC. Previous studies of concurrent chemoradiotherapy with cisplatin and 5-FU in patients with ESCC with T4 tumors and/or M1 LYM showed a clinical complete response rate of 15–33 % with a median PFS of 6 months and a 3-year overall survival rate of 23–26 % [3, 8, 9]. Our present study did not achieve the expected CR rate (39.4 %), but did demonstrate promising efficacy, with a median PFS of 12 months and a 3-year survival rate of 40.4 %.

The reason for not achieving the expected CR rate is unclear. The recent randomized phase 3 trials with induction chemotherapy for locally advanced head and neck squamous cell carcinoma showed survival benefits for DCF compared to CF, but the percentages of patients with a complete response and overall response did not statistically differ [12]. Patterns of failure in our present study showed that the distant recurrence rate was quite low (9 %). These might mean that this induction chemotherapy contributes to survival benefit by controlling minor metastases, but does not contribute to clinical response. The prolongation of PFS was clearly aided by the induction chemotherapy. Furthermore, post-protocol treatment, such as salvage surgery/endoscopic resection and palliative chemotherapy, might also have contributed to the regimen’s survival benefit. Only esophagectomy has curative potential, but salvage surgery is associated with high morbidity rates [17–19]. The participating centers in our present study are regarded as highly specialized, and complications of salvage surgery might therefore have been minimized. Salvage endoscopic resection has curative intent for patients with locoregional failure after definitive CRT for ESCC [20]. Moreover, salvage chemotherapy with taxanes has shown a clinical benefit for ESCC in patients who previously received platinum-based chemotherapy [21, 22]. While Ohtsu et al. [8] reported that survival rate with T4 disease was inferior to that with non-T4 disease, we saw no significant difference in response rate or survival benefit between our T4 and non-T4 groups, with an overall survival time of 26.0 versus 25.6 months, respectively.

Of the 13 patients who had no response after induction chemotherapy (clinical SD or PD), 2 achieved clinical CR and 4 achieved clinical PR after subsequent definitive CRT. A shift in strategy to definitive CRT might therefore be effective for non-responders to induction chemotherapy.

Although induction DCF induced severe neutropenia, as expected, febrile neutropenia occurred in only 6 % due to prophylactic use of ciprofloxacin. The risk of perforation of the esophageal wall related to definitive CRT has been highlighted in patients with T4 disease. Previous studies reported a frequency of perforation of the esophageal wall of 14–23 % in patients with T4 disease who received CRT [8, 9]. To reduce the risk of perforation, we decided to use induction chemotherapy before CRT, with the aim of decreasing tumor volume before encountering severe esophagitis. Treatment-related perforation of the esophageal wall occurred in only one patient during induction chemotherapy (3 %) and in a second patient during CRT (3 %). This incidence is lower than in the previous study [8, 9], suggesting that this strategy was effective, albeit with room for improvement. We did not mandate bronchoscopy to evaluate the degree of invasion of the trachea before treatment; doing so in T4 tracheal lesions before treatment will allow the prediction of perforation risk.

Induction with DCF followed by concurrent CRT using carboplatin was previously reported to have a CR rate of 16 % and median overall survival of 10.8 months [15]. Further, Higuchi et al. [23] reported that definitive CRT with DCF (DCF-R) for locally advanced ESCC had a high clinical CR rate (52.4 %) as well as prolonged PFS (median 11.1 months) and OS (MST 29.0 months). Although these survival data were equivalent to those of our present study, DCF-R was associated with a relatively high incidence of FN (grade 3 or more, 38.1 %) and late toxic effects, namely grade 3 or more pericardial effusion (2.6 %), esophagus-related toxicities (7.7 %) and cardiovascular toxicities (2.6 %). Accordingly, the use of induction chemotherapy followed by concurrent CRT or definitive DCF-R using three cytotoxic drugs for T4 and/or M1 LYM is still under discussion.

Several limitations of the study warrant mention. The total dose of irradiation for locally advanced ESCC is still not standardized. The Intergroup 0123 study found no improvement in survival or locoregional control when comparing a radiotherapy dose of 64.8 Gy with 50.4 Gy [24]. On this basis, the standard radiation dose for definitive CRT is now 50.0–50.4 Gy in the USA. In Japan, definitive chemoradiotherapy with ≥60 Gy has been employed to treat locally advanced ESCC, especially for T4 and/or M1 LYM [3, 8, 25]. Irradiation in our present study consisted of 60 Gy with a daily dose of 2 Gy. However, high-dose irradiation might increase the risk of perforation of the esophageal wall in the chemoradiation phase. Although the study included the efficacy and safety of induction DCF followed by CRT for patients with locally unresectable ESCC, it was conducted at expert centers in Japan. Thus, a conclusive answer to the optimum strategy for locally unresectable ESCC will require prospective randomized controlled trials at multiple institutions with a larger number of patients.

In conclusion, this study showed that induction chemotherapy with DCF followed by CRT was tolerable, and might feasible in patients with unresectable locally advanced ESCC.
